# Structural change detection in ordinal time series

**DOI:** 10.1371/journal.pone.0256128

**Published:** 2021-08-16

**Authors:** Fuxiao Li, Mengli Hao, Lijuan Yang

**Affiliations:** 1 Department of Applied Mathematics, Xi’an University of Technology, Xi’an, Shaanxi, People’s Republic of China; 2 Department of Mathematics and Information Science, Chang’an University, Xi’an, Shaanxi, People’s Republic of China; Normandie Universite, FRANCE

## Abstract

Change-point detection in health care data has recently obtained considerable attention due to the increased availability of complex data in real-time. In many applications, the observed data is an ordinal time series. Two kinds of test statistics are proposed to detect the structural change of cumulative logistic regression model, which is often used in applications for the analysis of ordinal time series. One is the standardized efficient score vector, the other one is the quadratic form of the efficient score vector with a weight function. Under the null hypothesis, we derive the asymptotic distribution of the two test statistics, and prove the consistency under the alternative hypothesis. We also study the consistency of the change-point estimator, and a binary segmentation procedure is suggested for estimating the locations of possible multiple change-points. Simulation results show that the former statistic performs better when the change-point occurs at the centre of the data, but the latter is preferable when the change-point occurs at the beginning or end of the data. Furthermore, the former statistic could find the reason for rejecting the null hypothesis. Finally, we apply the two test statistics to a group of sleep data, the results show that there exists a structural change in the data.

## Introduction

In categorical data analysis, ordinal categorical variables are frequently encountered in many contexts, such as health status (very good, good, so-so, bad, very bad), blood pressure (low, normal, high). The data observed hourly or daily constitutes an ordinal time series. The cumulative logistic regression model is often applied for analyzing the ordinal time series [1]. Sometimes the model may change at some unknown time moments (change-points) while it remains stable between these points. Structural stability is of prime importance in statistical modeling and inference. If the parameters have changed with the observed sample, inferences can be severely biased, and forecasts lose accuracy. Because of the importance of parameter stability, it is necessary to detect the structural change. Studies of structural change detection has been a popular research subject in statistics, see Csörgö and Horváth [2], Bai and Perron [3], Lee et al. [4], Perron [5], Gombay [6], Wang et al. [7], Chen et al. [8], Baranowski et al. [9], Wang et al. [10], Chen [11] and Liu et al. [12] for reviews of the field.

Structural changes detection in categorical data have been considered as well. Höhle [13] proposed a prospective CUSUM change-point detection procedure to detect a structural change in categorical time series; Wang et al. [10] described a procedure based on high-dimensional homogeneity test to detect and estimate multiple change-point in multinomial data; Plasse and Adams [14] illustrated a multiple change-point detection method for categorical data streams, which could adaptively monitor the category probabilities. As generalized linear regression models for categorical time series allow for parsimonious modeling and incorporation of random time-dependent covariates, Fokianos and Kedem [15] suggested the generalized linear model for categorical time series modeling. For change-point detection in the generalized linear model, Xia et al. [16] introduced two procedures to sequentially detect the structural change in generalized linear models with assuming independence; Hudecová [17] investigated the detection of change in autoregressive models for binary time series; Fokianos et al. [18] provided a statistical procedure based on the partial likelihood score process to detect a structural change in binary logistic regression model; Gombay et al. [19] and Li et al. [20] discussed retrospective change detection and sequential change detection in multinominal logistic regression model.

Score test for detection of changes in time series models has been studied by Gombay and Serban [21], Gombay et al. [22]. The test statistic is usually computationally less demanding than the likelihood ratio test statistic. In this paper, we first propose a test statistic based on the efficient score vector to detect a structural change in cumulative logistic regression model, which extends the change-point detection of Gombay et al. [19]. Simulation shows that the empirical power of the proposed statistic is low when the change-point occurs at the beginning or end of the data. To this end, we propose a new statistic, which is the quadratic form of the efficient score vector and has a weight function. Under the null hypothesis of no change, we derive the asymptotic distribution of the two statistics, and prove the consistency under the alternative hypothesis. We also study the consistency of the change-point estimator, and a binary segmentation procedure is suggested for estimating the locations of possible multiple change-points. Simulation results show that the empirical size of the two statistics is close to the significance level 0.05, and the empirical power is approximate to 1 when the sample size is large. The empirical power of the former statistic is higher when the change-point is located at the centre of the data, but the latter performs better when the change-point is located at the beginning or end of the data. Furthermore, the former statistic could find the reason for rejecting the null hypothesis. Finally, we apply the two statistics to study a group of sleep data, and find a structural change in the data.

## The model and hypotheses

Consider a categorical time series {***Y***_*t*_} with *m* categories, ***Y***_*t*_ = (*Y*_*t*1_, …, *Y*_*tq*_)′, *q* = *m* − 1,

$$Y_{tj}=\{\begin{array}{cc} 1, & \text{if}\;\text{the}\;j\;\;\text{category}\;\text{is}\;\text{observed}\;\text{at}\;\text{time}\;t,\\ 0, & \text{otherwise} \end{array}$$

for *t* = 1, 2, …, *n* and *j* = 1, …, *q*, $Y_{tm}=1-\sum_{j=1}^{q}Y_{tj}$. The vector of conditional probability ***π***_*t*_ = (*π*_*t*1_, …, *π*_*tq*_)′ is defined by

$$\pi_{tj}=E(Y_{tj}|F_{t-1})=P(Y_{tj}=1|F_{t-1}),\;\;j=1,\ldots ,q,$$

for every *t*, $\pi_{tm}=1-\sum_{j=1}^{q}\pi_{tj}$, where

$$F_{t-1}=\sigma \{Y_{t-1},Y_{t-2},\ldots ,Z_{t-1},Z_{t-2},\ldots \},$$

{***Z***_*t*−1_} denotes the *p* × *q* covariate matrices.

Define an ordinal time series {*Y*_*t*_}, where *Y*_*t*_ = *j* is equivalent to *Y*_*tj*_ = 1 for *j* = 1, 2, …, *m*, *t* = 1, 2, …, *n*. Let {*X*_*t*_} be a latent variable time series, where *X*_*t*_ = −***β***′***z***_*t*−1_ + *e*_*t*_, ***β*** ∈ ***R***^*d*^, ***z***_*t*−1_ is a *d*-dimensional covariate vector, *e*_*t*_ is a white noise process with continuous cumulative distribution function *F*. Suppose that −∞ = *α*_0_ < *α*_1_ < ⋯ < *α*_*m*_ = ∞ are threshold parameters, such that *Y*_*t*_ satisfies

$$Y_{t}=j\;\text{if}\;\alpha_{j-1}\leq X_{t}<\alpha_{j}$$

for *j* = 1, 2, …, *m*. According to the equivalence relation between *Y*_*t*_ and *Y*_*tj*_, we have

$$\begin{array}{ccc} \pi_{tj} & = & P(Y_{tj}=1|F_{t-1})=P(Y_{t}=j|F_{t-1})=P(\alpha_{j-1}\leq X_{t}<\alpha_{j}|F_{t-1})\\ & = & F(\alpha_{j}+\beta^{\prime }z_{t-1})-F(\alpha_{j-1}+\beta^{\prime }z_{t-1}), \end{array}$$

then

$$P(Y_{t}\leq j|F_{t-1})=F(\alpha_{j}+\beta^{\prime }z_{t-1}),\;j=1,2,\ldots ,m.$$

If *F*(*x*) is the logistic distribution function, then *F*^−1^ is the logistic link function log *it*(*x*), where logit (*x*) = ln (*x*/(1 − *x*)), 0 < *x* < 1. Thus we have

$$\log it(P(Y_{t}\leq j|F_{t-1}))=\alpha_{j}+\beta^{\prime }z_{t-1},\;j=1,2,\ldots ,q,$$

which is called the cumulative logistic regression model.

Let ***θ*** = (*α*_1_, …, *α*_*q*_, ***β***′)′ be a *p*-dimensional parameter vector, *p* = *q* + *d*. In this paper we wish to test if there exists a structural change in the parameter ***θ***, that is,

$$\begin{array}{ccc} & & H_{0}:\theta =\theta_{0},\;t=1,2,\ldots ,n,\\ & & H_{A}:\theta =\theta_{0},\;t=1,2,\ldots ,k^{*},\;\text{and}\;\theta =\theta_{0}^{*},\;t=k^{*}+1,\ldots ,n, \end{array}$$

where ***θ***_0_ is the true value of ***θ***, k* denotes the change-point which occurs in some of the parameter ***θ***, $\theta_{0}\neq \theta_{0}^{*}$, ***θ***_0_, $\theta_{0}^{*}$ and k* are unknown.

Next, we estimate the parameter vector ***θ*** by the partial likelihood method (Fokianos et al. [18]). The partial likelihood function

$$PL(\theta )=\prod_{t=1}^{n}f(Y_{t};\theta |F_{t-1})=\prod_{t=1}^{n}\prod_{j=1}^{m}\pi_{tj}^{Y_{tj}}(\theta )$$

and the partial log-likelihood function

$$\begin{array}{c} l(\theta )=\log PL(\theta )=\sum_{t=1}^{n}\sum_{j=1}^{m}Y_{tj}\log\pi_{tj}(\theta ) \end{array}$$ (1)

are defined in Gombay et al. [19]. Denote the partial score vector

$$\begin{array}{ccc} S_{n}(\theta ) & = & \nabla l(\theta )={\left(\frac{\partial l(\theta )}{\partial \theta_{1}},\ldots ,\frac{\partial l(\theta )}{\partial \theta_{p}}\right)}^{\prime }\\ & = & \sum_{t=1}^{n}Z_{t-1}U_{t}(\theta )(Y_{t}-\pi_{t}(\theta ))=\sum_{t=1}^{n}Z_{t-1}D_{t}(\theta )\Sigma_{t}^{-1}(\theta )(Y_{t}-\pi_{t}(\theta )), \end{array}$$

where

$$Z_{t-1}={\left(\begin{array}{cccc} 1 & 0 & \cdots & 0\\ 0 & 1 & \cdots & 0\\ \vdots & \vdots & & \vdots \\ \begin{array}{c} 0\\ z_{t-1} \end{array} & \begin{array}{c} 0\\ z_{t-1} \end{array} & \begin{array}{c} \cdots \\ \cdots \end{array} & \begin{array}{c} 0\\ z_{t-1} \end{array} \end{array}\right)}_{p\times q},$$

$U_{t}(\theta )=D_{t}(\theta )\Sigma_{t}^{-1}(\theta )$, $D_{t}(\theta )=\frac{\partial h(\eta_{t})}{\partial \eta_{t}^{\prime }}$, ***h***(***η***_*t*_) = (*h*_1_(***η***_*t*_), …, *h*_*q*_(***η***_*t*_))′, $\eta_{t}=Z_{t-1}^{\prime }\theta ={\left(\eta_{t1},\ldots ,\eta_{tq}\right)}^{\prime }$. *h*_1_(***η***_*t*_), …, *h*_*q*_(***η***_*t*_) satisfies

$$\begin{array}{ccc} & & \pi_{t1}(\theta )=h_{1}(\eta_{t1})=F(\eta_{t1}),\ldots ,\\ & & \pi_{tj}(\theta )=h_{j}(\eta_{t})=F(\eta_{tj})-F(\eta_{t(j-1)}),j=2,\ldots ,q, \end{array}$$

where $F(x)=\frac{1}{1+\exp(-x)}$. **Σ**_*t*_(***θ***) is the conditional covariance matrix of ***Y***_*t*_ with

$$\begin{array}{c} \Sigma_{t}^{\left(i,j\right)}(\theta )=\{\begin{array}{cc} -\pi_{ti}(\theta )\pi_{tj}(\theta ), & i\neq j,\\ \pi_{ti}(\theta )(1-\pi_{ti}(\theta )), & i=j, \end{array} \end{array}$$

for *i*, *j* = 1, …, *q* [23].

To obtain the existence, consistency and asymptotic normality of the maximum partial likelihood estimator, we give a few assumptions on the the covariate matrices {***Z***_*t*_} and parameter vector ***θ***.

**Assumption 1***The parameter vector*$\theta \in \Omega \subseteq R^{p}$, *where* Ω *is an open set*.

**Assumption 2***The link function h is twice continuously differentiable, and satisfies* det(*∂* *h*(***η***_*t*_)/*∂* ***η***_*t*_) ≠ 0, *where* $\eta_{t}=Z_{t-1}^{\prime }\theta$.

**Assumption 3***The covariate matrix****Z***_*t*−1_*lies almost surely in a non-random compact subset***Φ** of $R^{p\times q}$ *such that* $P(\lambda^{\prime }(\sum_{t=1}^{n}Z_{t-1}Z_{t-1}^{\prime })\lambda >0)=1$, $Z_{t-1}^{\prime }\theta$ *lies almost surely in the domain H of h for all* ***Z***_*t*−1_ ∈ **Φ** and ***θ*** ∈ **Ω**, *where* $\lambda \in R^{p}$, **λ** ≠ ***0***.

Assumptions 1 and 2 ensure that the second derivative of *l*(***θ***) is continuous, det(*∂* ***h***(***η***_*t*_)/*∂* ***η***_*t*_) ≠ 0 implies that ***U***_*t*_(***θ***) is not singular (Fokianos and Kedem [24]). From Assumption 3,

$$G_{n}(\theta )=\sum_{t=1}^{n}Z_{t-1}U_{t}(\theta )\Sigma_{t}(\theta )U_{t}^{\prime }(\theta )Z_{t-1}^{\prime }.$$

is positive definite with probability one [24]. Since the likelihood estimation employs an assumption regarding ergodicity of the joint process ${\left(Y_{t}^{T},z_{t}^{T}\right)}^{T}$ (Fokianos and Truquet [25]), let {*Y*_*t*_} be a time series taking values in a finite set *E* with cardinal *m*, and such that

$$P(Y_{t}=\omega |Y_{t-1}^{-},z)=q(\omega |Y_{t-1}^{-},z_{t-1}^{-}),t\in Z,$$

where $z=\{z_{t},t\in Z\}$, $Y_{t-1}^{-}=\{Y_{t-1-j},j\geq 0\}$, $z_{t-1}^{-}=\{z_{t-1-j},j\geq 0\}$, *q* is a transition kernel. We assume that the applications $(\omega ,y,x)|\to q(\omega |y,x_{t-1}^{-})$ are measurable, as applications from $E\times E^{m}\times D$ to (0, 1), where $\left\{x_{t},t\in Z\right\}$ is a sequence, $x_{t-1}^{-}=\{x_{t-1-j},j\geq 0\}$, $D\in B{\left(R^{d}\right)}^{⊗Z}$ is such that P(z∈D)=1. Assume that *v*_1_ and *v*_2_ are two probability measures on *E*, define

$$d_{TV}(v_{1},v_{2})=\frac{1}{2}\sum_{f\in E}|v_{1}(f)-v_{2}(f)|.$$

For *y*, *y*′ ∈ *E*^*m*^ and a positive integer *s*, we write $y\overset{s}{=}y^{\prime }$ if $y_{i}=y_{i}^{\prime }$, 0 ≤ *i* ≤ *s* − 1 (Truquet [26]).

**Assumption 4***The d-dimensional covariate vector* {***z***_*t*−1_} *is stationary and ergodic*.

**Assumption 5***Setting for s* ≥ 0,

$$b_{s}=\sup\{d_{TV}(q(\cdot |y,x_{t}^{-}),q(\cdot |y^{\prime },x_{t}^{-})):(y,y^{\prime },x)\in E^{m}\times E^{m}\times D,t\in Z,y\overset{s}{=}y^{\prime }\},$$

*we have* *b*_0_ < 1 and $\sum_{s\geq 0}b_{s}<\infty$.

Assumptions 4 and 5 guarantees that ${\left(Y_{t}^{T},z_{t}^{T}\right)}^{T}$ is stationary and ergodic [26]. Assumptions 1–5 are required to obtain consistency and asymptotic normality of the maximum likelihood estimator. However, existence of moments for the covariate process is still required to study large sample properties of the maximum likelihood estimator [25]. So we have

**Assumption 6**$E{\left|z_{t-1}^{\left(i\right)}\right|}^{4}<\infty$, *i* = 1, 2, ⋯, *d*, *where* $z_{t-1}^{\left(i\right)}$, 1 ≤ *i* ≤ *d are components of vector* ***z***_*t*‒1_.

## The proposed testing procedure

Based on the partial likelihood score process, a test statistic is defined by

$$W_{1}=\underset{1\leq k\leq n}{\text{max}}n^{-1/2}{\hat{T}}_{n}^{-1/2}S_{k}({\hat{\theta }}_{n})$$

where ${\hat{T}}_{n}=\frac{1}{n}S_{n}\left({\hat{\theta }}_{n}\right)S_{n}^{\prime }\left({\hat{\theta }}_{n}\right)$, ${\hat{\theta }}_{n}$ is the maximum partial likelihood estimator of *θ*, which can be obtained by maximizing the partial log-likelihood function (1) (see Fokianos and Kedem [23]).

Under the null hypothesis of no change, we derive the asymptotic distribution of the proposed test statistic.

**Theorem 1***If Assumptions 1–6 and H*_0_*hold, then we have*

$$W_{1}=\underset{1\leq k\leq n}{\text{max}}n^{-1/2}{\hat{T}}_{n}^{-1/2}S_{k}({\hat{\theta }}_{n})\overset{d}{\to }\underset{0<t<1}{\text{sup}}B\left(t\right),$$

*where*${\hat{T}}_{n}=\frac{1}{n}S_{n}\left({\hat{\theta }}_{n}\right)S_{n}^{\prime }\left({\hat{\theta }}_{n}\right)$, ***B***(*t*) *is a p-dimensional vector of independent Brownian bridge*, $\overset{d}{\to }$*means convergence in distribution*.

**Proof**: Since $S_{n}\left({\hat{\theta }}_{n}\right)=0$, we can write

$$n^{-1/2}\left(S_{k}\left({\hat{\theta }}_{n}\right)\right)=n^{-1/2}\left(S_{k}\left({\hat{\theta }}_{n}\right)-\frac{k}{n}S_{n}\left({\hat{\theta }}_{n}\right)\right),$$

let $S_{k}^{\left(i\right)}$ denote the *i*-th element of ***S***_*k*_, *i* = 1, 2, …, *p*, ***θ***_0_ is the true value of ***θ***, then we have

$$\begin{array}{ccc} & & n^{-1/2}S_{k}^{\left(i\right)}({\hat{\theta }}_{n})=n^{-1/2}(S_{k}^{\left(i\right)}({\hat{\theta }}_{n})-\frac{k}{n}S_{n}^{\left(i\right)}({\hat{\theta }}_{n}))\\ & & =n^{-1/2}(S_{k}^{\left(i\right)}(\theta_{0})-\frac{k}{n}S_{n}^{\left(i\right)}(\theta_{0}))-n^{1/2}\sum_{j=1}^{p}({\hat{\theta }}_{n}^{\left(j\right)}-\theta_{0}^{\left(j\right)})\\ & & \times \frac{1}{n}(\sum_{t=1}^{k}{\left(Z_{t-1}U_{t}(\theta_{0})(Y_{t}-\pi_{t}(\theta_{0}))\right)}^{\left(i\right)}{\left(Z_{t-1}U_{t}(\theta_{0})(Y_{t}-\pi_{t}(\theta_{0}))\right)}^{\left(j\right)}\\ & & -\frac{k}{n}\sum_{t=1}^{n}{\left(Z_{t-1}U_{t}(\theta_{0})(Y_{t}-\pi_{t}(\theta_{0}))\right)}^{\left(i\right)}{\left(Z_{t-1}U_{t}(\theta_{0})(Y_{t}-\pi_{t}(\theta_{0}))\right)}^{\left(j\right)})+E_{kn}^{\left(i\right)}. \end{array}$$

Next, it is similar to the proof of Proposition 3 in Gombay et al. [19], we can prove that

$$\begin{array}{ccc} & & \max_{1\leq k\leq n}\frac{1}{n}|\sum_{t=1}^{k}{\left(Z_{t-1}U_{t}\left(\theta_{0}\right)\left(Y_{t}-\pi_{t}\left(\theta_{0}\right)\right)\right)}^{\left(i\right)}{\left(Z_{t-1}U_{t}\left(\theta_{0}\right)\left(Y_{t}-\pi_{t}\left(\theta_{0}\right)\right)\right)}^{\left(j\right)}\\ & & -\frac{k}{n}\sum_{t=1}^{n}{\left(Z_{t-1}U_{t}\left(\theta_{0}\right)\left(Y_{t}-\pi_{t}\left(\theta_{0}\right)\right)\right)}^{\left(i\right)}{\left(Z_{t-1}U_{t}\left(\theta_{0}\right)\left(Y_{t}-\pi_{t}\left(\theta_{0}\right)\right)\right)}^{\left(j\right)}|=o_{P}\left(1\right) \end{array}$$

By Theorem 4.1 of Fokianos and Kedem [23], we get

$$n^{1/2}\left({\hat{\theta }}_{n}^{\left(i\right)}-\theta_{0}^{\left(i\right)}\right)=O_{P}\left(1\right).$$

The error terms $E_{kn}^{\left(i\right)}$ have higher orders of products of $\left({\hat{\theta }}_{n}^{\left(i\right)}-\theta_{0}^{\left(i\right)}\right)$, it can be shown that $E_{kn}^{\left(i\right)}=o_{P}\left(1\right)$. According to Proposition 1 (Gombay et al. [19]) and Slutsky’s theorem, we get

$$\max_{1\leq k\leq n}n^{-1/2}{\hat{T}}_{n}^{-1/2}S_{k}\left({\hat{\theta }}_{n}\right)\overset{d}{\to }\underset{0<t<1}{\sup}B\left(t\right)$$

as *n* → ∞.

**Remark 1** When using the above test, if there exists some *i*, 1 ≤ *i* ≤ *p*,

$$\begin{array}{c} \max_{1\leq k\leq n}n^{-1/2}|{\left({\hat{T}}^{-1/2}S_{k}\left({\hat{\theta }}_{n}\right)\right)}^{\left(i\right)}|\geq C\left(\alpha^{*}\right), \end{array}$$

the null hypothesis is rejected and a change-point occurs, α* = 1 ‒ (1 ‒ *α*)^1/*p*^. Let *B*(*u*) be a one-dimensional Brownian bridge, Csörgö and Révész [27] suggested that *C*(α*) could be obtained by

$$P\left(\underset{0\leq u\leq 1}{\sup}|B\left(u\right)|\geq x\right)=\sum_{k\neq 0}{\left(-1\right)}^{k+1}\exp\left(-2k^{2}x^{2}\right).$$

Simulation shows that *W*_1_ has poor performance at the boundaries. In particular, the limiting Brownian bridge is tied down at *t* = 0 and *t* = 1 (meaning *B*(0) = *B*(1) = 0), and hampers the ability of the test to detect the structural change occurring near the beginning or end of the data. Many authors address this problem by adding a weight function [28]. Therefore, we construct a new test statistic

$$W_{2}=\underset{l<\frac{k}{n}<h}{\text{max}}\frac{n^{-1}\xi_{k}^{\prime }\xi_{k}}{k/n(1-k/n)},$$

which is the quadratic form of the efficient score vector and has a weight function, where

$$\xi_{k}={\left({\left({\hat{T}}_{n}^{-1/2}S_{k}\left({\hat{\theta }}_{n}\right)\right)}^{\left(i_{1}\right)},\ldots ,{\left({\hat{T}}_{n}^{-1/2}S_{k}\left({\hat{\theta }}_{n}\right)\right)}^{\left(i_{j}\right)}\right)}^{\prime },$$

{*i*_1_, *i*_2_, …, *i*_*j*_} ⊂ {1, 2, …, *p*}, *j* = 1, 2, …, *p*, 0 < *l* < *h* < 1.

**Theorem 2***If Assumptions 1–6 and H*_0_*hold, then we have*

$$W_{2}=\max_{l<\frac{k}{n}<h}\frac{n^{-1}\xi_{k}^{\prime }\xi_{k}}{k/n(1-k/n)}\overset{d}{\to }\underset{l<t<h}{\sup}\frac{\sum_{i=1}^{j}B_{i}^{2}\left(t\right)}{t\left(1-t\right)}$$

*for each* 0 < *l* < *h* < 1, *B*_*i*_(*t*), *i* = 1, …, *j* *are independent one-dimensional Brownian bridges*.

The conclusion of Theorem 2 can be deduced directly from Theorem 1. To obtain the critial values of the asymptotic distribution, Csörgö and Horváth [2] used a result of Vostrikova [29] to show that

$$P\{\underset{l<t<h}{\sup}\frac{\sum_{i=1}^{j}B_{i}^{2}\left(t\right)}{t\left(1-t\right)}\geq x\}=\frac{x^{j/2}e^{-x/2}}{2^{j/2}\Gamma \left(j/2\right)}\{\left(1-\frac{j}{x}\right)\log\frac{\left(1-l\right)h}{l\left(1-h\right)}+\frac{4}{x}+O\left(\frac{1}{x^{2}}\right)\}$$

as *x* → ∞. For example, when *α* = 0.05, *l* = 0.05, *h* = 0.95, *j* = 2, the critical value *C*(*α*) = 13.1.

Under the alternative hypothesis, there exists a structural change in the model, then we will prove the consistency of the two statistics.

**Theorem 3***Suppose Assumptions 1–6 and H*_*A*_*hold, if the coefficient changes from****θ***_0_*to*$\theta_{0}^{*}$atk*, $\theta_{0}^{*\left(j\right)}=\theta_{0}^{\left(j\right)}+\delta$, $\theta_{0}^{\left(j\right)}$*is the jth component of****θ***_0_, *j* ∈ {1, 2, …, *p*}, *where* *δ* *is a constant*, *δ* ≠ 0, *then we have*

(i)
$$W_{1}=\max_{1\leq k\leq n}n^{-1/2}\|{\hat{T}}_{n}^{-1/2}S_{k}\left({\hat{\theta }}_{n}\right)\|\overset{P}{\to }\infty ;$$
(ii)
$$W_{2}=\max_{l<\frac{k}{n}<h}\frac{n^{-1}\xi_{k}^{\prime }\xi_{k}}{k/n(1-k/n)}\overset{P}{\to }\infty ,$$

where 0 < *l* < *h* < 1, ‖⋅‖ *denotes the Euclidean norm of a vector*, $\overset{P}{\to }$ *means convergence in probability*.

**Proof**: Under the alternative hypothesis ***θ*** = ***θ***_0_, *t* = 1, 2, …, k*, $\theta \;=\;\theta_{0}^{*}$, *t* = k* + 1, …, *n*. Suppose that the coefficient changes from ***θ***_0_ to $\theta_{0}^{*}$ at k*, $\theta_{0}^{*\left(j\right)}=\theta_{0}^{\left(j\right)}+\delta$, $\theta_{0}^{\left(j\right)}$ is the *j*-th component of ***θ***_0_, 1 < *j* < *p*, where *δ* is a constant, *δ* ≠ 0.

When k* < *k* < *n*,

$$n^{-1/2}S_{k}\left({\hat{\theta }}_{n}\right)=n^{-1/2}\left(S_{1k}\left({\hat{\theta }}_{n}\right)+S_{2k}\left({\hat{\theta }}_{n}\right)\right),$$

where $S_{1k}=\sum_{t=1}^{k^{*}}Z_{t-1}U_{t}\left({\hat{\theta }}_{n}\right)\left(Y_{t}-\pi_{t}\left({\hat{\theta }}_{n}\right)\right)$, $S_{2k}=\sum_{t=k^{*}+1}^{k}Z_{t-1}U_{t}\left({\hat{\theta }}_{n}\right)\left(Y_{t}-\pi_{t}\left({\hat{\theta }}_{n}\right)\right)$. For the *i*th component of $S_{1k}\left({\hat{\theta }}_{n}\right)$, 1 < *i* < *p*, we have

$$\begin{array}{ccc} & & n^{-1/2}S_{1k}^{\left(i\right)}\left({\hat{\theta }}_{n}\right)=n^{-1/2}S_{1k}^{\left(i\right)}\left(\theta_{0}\right)+n^{1/2}\sum_{j=1}^{p}\left({\hat{\theta }}_{n}^{\left(j\right)}-\theta_{0}^{\left(j\right)}\right)\\ & & \times \frac{1}{n}\sum_{t=1}^{k^{*}}{\left(Z_{t-1}U_{t}\left(\theta_{0}\right)\left(Y-\pi_{t}\left(\theta_{0}\right)\right)\right)}^{\left(i\right)}{\left(Z_{t-1}U_{t}\left(\theta_{0}\right)\left(Y-\pi_{t}\left(\theta_{0}\right)\right)\right)}^{\left(j\right)}+E_{1k}^{\left(i\right)}\\ & & n^{-1/2}S_{2k}^{\left(i\right)}\left({\hat{\theta }}_{n}\right)=n^{-1/2}S_{2k}^{\left(i\right)}\left(\theta_{0}^{*}\right)+n^{1/2}\sum_{j=1}^{p}\left({\hat{\theta }}_{n}^{\left(j\right)}-\theta_{0}^{*\left(j\right)}\right)\\ & & \times \frac{1}{n}\sum_{t=k^{*}+1}^{k}{\left(Z_{t-1}U_{t}\left(\theta_{0}^{*}\right)\left(Y-\pi_{t}\left(\theta_{0}^{*}\right)\right)\right)}^{\left(i\right)}{\left(Z_{t-1}U_{t}\left(\theta_{0}^{*}\right)\left(Y-\pi_{t}\left(\theta_{0}^{*}\right)\right)\right)}^{\left(j\right)}+E_{2k}^{\left(i\right)} \end{array}$$

where $E_{1k}^{\left(i\right)}$ has two orders of products of $\left({{\hat{\theta }}_{n}}^{\left(j\right)}-\theta_{0}^{\left(j\right)}\right)$, $E_{2k}^{\left(i\right)}$ has two orders of products of $\left({\hat{\theta }}_{n}^{\left(j\right)}-\theta_{0}^{*\left(j\right)}\right)$. By Theorem 1 we have

$$n^{-1/2}S_{2k}^{\left(i\right)}\left(\theta_{0}^{*}\right)=O_{P}\left(1\right)$$

as *n* → ∞. Following Assumptions 1–6, we conclude that

$$\frac{1}{n}\sum_{t=k^{*}+1}^{k}{\left(Z_{t-1}U_{t}\left(\theta_{0}^{*}\right)\left(Y-\pi_{t}\left(\theta_{0}^{*}\right)\right)\right)}^{\left(i\right)}{\left(Z_{t-1}U_{t}\left(\theta_{0}^{*}\right)\left(Y-\pi_{t}\left(\theta_{0}^{*}\right)\right)\right)}^{\left(j\right)}=O_{P}\left(1\right).$$

Since *δ* ≠ 0, we have

$$n^{1/2}\left({\hat{\theta }}_{n}^{\left(j\right)}-\theta_{0}^{*\left(j\right)}\right)=n^{1/2}\left({\hat{\theta }}_{n}^{\left(j\right)}-\theta_{0}^{\left(j\right)}-\delta \right)\overset{P}{\to }\infty$$

as *n* → ∞. When 1 < *k* < k*, the proof is similar. The proof of (ii) is similar to the proof of (i).

Once the null hypothesis is rejected, indicating there may exist a change-point, then we locate the change-point position by

$$\begin{array}{c} {\hat{k}}^{*}=\min\{k:\max_{1\leq k\leq n}n^{-1/2}|{\left({\hat{T}}_{n}^{-1/2}S_{k}\left({\hat{\theta }}_{n}\right)\right)}^{\left(i\right)}|,1\leq i\leq p\}. \end{array}$$ (2)

The following theorem shows that the change-point estimator ${\hat{k}}^{*}$ is consistent for the true change-point k*, as *n* → ∞.

**Theorem 4**Let k* *be the true position of change-point under the alternative hypothesis H*_*A*_ and ${\hat{k}}^{*}$ be the estimate of k* *given by* (2). *Under Assumptions 1–6, then* ${\hat{k}}^{*}$ *is consistent to* k*, as *n* → ∞.

**Proof**: First we note that

$$\begin{array}{ccc} & & n^{-1/2}S_{k}^{\left(i\right)}({\hat{\theta }}_{n})=n^{-1/2}(S_{k}^{\left(i\right)}({\hat{\theta }}_{n})-\frac{k}{n}S_{n}^{\left(i\right)}({\hat{\theta }}_{n}))\\ & & =n^{-1/2}(S_{k}^{\left(i\right)}(\theta_{0})-\frac{k}{n}S_{n}^{\left(i\right)}(\theta_{0}))-n^{1/2}\sum_{j=1}^{q+d}({\hat{\theta }}_{n}^{\left(j\right)}-\theta_{0}^{\left(j\right)})\\ & & \times \frac{1}{n}(\sum_{t=1}^{k}{\left(Z_{t-1}U_{t}(\theta_{0})(Y_{t}-\pi_{t}(\theta_{0}))\right)}^{\left(i\right)}{\left(Z_{t-1}U_{t}(\theta_{0})(Y_{t}-\pi_{t}(\theta_{0}))\right)}^{\left(j\right)}\\ & & -\frac{k}{n}\sum_{t=1}^{n}{\left(Z_{t-1}U_{t}(\theta_{0})(Y_{t}-\pi_{t}(\theta_{0}))\right)}^{\left(i\right)}{\left(Z_{t-1}U_{t}(\theta_{0})(Y_{t}-\pi_{t}(\theta_{0}))\right)}^{\left(j\right)})+E_{kn}^{\left(i\right)}, \end{array}$$

where *i* = 1, 2, …, *p*. Since

$$S_{k}^{\left(i\right)}\left(\theta_{0}\right)-\frac{k}{n}S_{n}^{\left(i\right)}\left(\theta_{0}\right)=\frac{k\left(n-k\right)}{n}\left(\frac{S_{k}^{\left(i\right)}\left(\theta_{0}\right)}{k}-\frac{S_{n-k}^{\left(i\right)}\left(\theta_{0}\right)}{n-k}\right),$$

where $S_{n-k}\left(\theta_{0}\right)=\sum_{t=k+1}^{n}Z_{t-1}U_{t}\left(\theta_{0}\right)\left(Y_{t}-\pi_{t}\left(\theta_{0}\right)\right)$. And because

$$\begin{array}{c} E\left(Y_{tj}|F_{t-1}\right)=\pi_{tj}\left(\theta_{0}\right),t=1,2,\cdots ,k^{*},j=1,2,\cdots ,m-1,\\ E\left(Y_{tj}|F_{t-1}\right)=\pi_{tj}\left(\theta_{0}^{*}\right),t=k^{*}+1,k^{*}+2,\cdots ,n,j=1,2,\cdots ,m-1, \end{array}$$

Therefore $E\left(S_{k}^{\left(i\right)}\left(\theta_{0}\right)-\frac{k}{n}S_{n}^{\left(i\right)}\left(\theta_{0}\right)\right)$ increases as *k* = 1, 2, ⋯, k*, and decrease as *k* = k* + 1, k* + 2, ⋯, *n*, then we take (2) as the change-point estimator.

By the proof of Theorem 1, we have

$$\begin{array}{ccc} & & -n^{1/2}\sum_{j=1}^{p}({\hat{\theta }}_{n}^{\left(j\right)}-\theta_{0}^{\left(j\right)})\\ & & \times \frac{1}{n}(\sum_{t=1}^{k}{\left(Z_{t-1}U_{t}(\theta_{0})(Y_{t}-\pi_{t}(\theta_{0}))\right)}^{\left(i\right)}{\left(Z_{t-1}U_{t}(\theta_{0})(Y_{t}-\pi_{t}(\theta_{0}))\right)}^{\left(j\right)}\\ & & -\frac{k}{n}\sum_{t=1}^{n}{\left(Z_{t-1}U_{t}(\theta_{0})(Y_{t}-\pi_{t}(\theta_{0}))\right)}^{\left(i\right)}{\left(Z_{t-1}U_{t}(\theta_{0})(Y_{t}-\pi_{t}(\theta_{0}))\right)}^{\left(j\right)})=o_{P}(1) \end{array}$$

and $E_{kn}^{\left(i\right)}=o_{P}\left(1\right)$. By Theorem 2 of Gombay [6], to prove (2) it is enough to show that

$$\begin{array}{c} \lim_{K\to \infty }\underset{n\to \infty }{\lim\sup}P\{\max_{1<k\leq k^{*}-K}f_{k}\geq \max_{k^{*}-K<k\leq k^{*}+K}f_{k}\}=0, \end{array}$$ (3)

and

$$\begin{array}{c} \lim_{K\to \infty }\underset{n\to \infty }{\lim\sup}P\{\max_{k^{*}+K<k\leq n}f_{k}\geq \max_{k^{*}-K<k\leq k^{*}+K}f_{k}\}=0, \end{array}$$ (4)

where $f_{k}=n^{-1/2}\left(S_{k}^{\left(i\right)}\left({\hat{\theta }}_{n}\right)-\frac{k}{n}S_{n}^{\left(i\right)}\left({\hat{\theta }}_{n}\right)\right)$. To prove (3), assume that there exists a constant *K*, *K* < k*,

$$\begin{array}{ccc} & & P\{\max_{1<k\leq k^{*}-K}n^{-1/2}\left(S_{k}^{\left(i\right)}\left({\hat{\theta }}_{n}\right)-\frac{k}{n}S_{n}^{\left(i\right)}\left({\hat{\theta }}_{n}\right)\right)\\ & & >\max_{k^{*}-K<k\leq k^{*}+K}n^{-1/2}\left(S_{k}^{\left(i\right)}\left({\hat{\theta }}_{n}\right)-\frac{k}{n}S_{n}^{\left(i\right)}\left({\hat{\theta }}_{n}\right)\right)\}\\ & & \leq P\{\max_{1<k\leq k^{*}-K}n^{-1/2}\left(S_{k}^{\left(i\right)}\left({\hat{\theta }}_{n}\right)-\frac{k}{n}S_{n}^{\left(i\right)}\left({\hat{\theta }}_{n}\right)\right)\\ & & >\max_{k^{*}-K<k\leq k^{*}}n^{-1/2}\left(S_{k}^{\left(i\right)}\left({\hat{\theta }}_{n}\right)-\frac{k}{n}S_{n}^{\left(i\right)}\left({\hat{\theta }}_{n}\right)\right)\}\\ & & =P\{\exists k:1<k\leq k^{*}-K,\max_{1\leq r\leq k^{*}-k}n^{-1/2}\left(S_{r}^{\left(i\right)}\left({\hat{\theta }}_{n}\right)-\frac{r}{n}S_{n}^{\left(i\right)}\left({\hat{\theta }}_{n}\right)\right)\leq 0\}\\ & & \leq P\{n^{-1/2}\left(S_{K}^{\left(i\right)}\left({\hat{\theta }}_{n}\right)-\frac{K}{n}S_{n}^{\left(i\right)}\left({\hat{\theta }}_{n}\right)\right)<0\}\\ & & =P\{n^{-1/2}\left(S_{K}^{\left(i\right)}\left(\theta_{0}\right)-\frac{K}{n}S_{n}^{\left(i\right)}\left(\theta_{0}\right)\right)+S_{K}^{*\left(i\right)}<0\}, \end{array}$$

where

$$\begin{array}{ccc} & & S_{K}^{*\left(i\right)}=-n^{1/2}\sum_{j=1}^{p}({\hat{\theta }}_{n}^{\left(j\right)}-\theta_{0}^{\left(j\right)})\\ & & \times \frac{1}{n}(\sum_{t=1}^{k}{\left(Z_{t-1}U_{t}(\theta_{0})(Y_{t}-\pi_{t}(\theta_{0}))\right)}^{\left(i\right)}{\left(Z_{t-1}U_{t}(\theta_{0})(Y_{t}-\pi_{t}(\theta_{0}))\right)}^{\left(j\right)}\\ & & -\frac{k}{n}\sum_{t=1}^{n}{\left(Z_{t-1}U_{t}(\theta_{0})(Y_{t}-\pi_{t}(\theta_{0}))\right)}^{\left(i\right)}{\left(Z_{t-1}U_{t}(\theta_{0})(Y_{t}-\pi_{t}(\theta_{0}))\right)}^{\left(j\right)})+E_{kn}^{\left(i\right)}=o_{P}(1). \end{array}$$

By Theorem 1, choosing *δ* > 0 arbitrarily

$$\lim_{n\to \infty }P\{n^{-1/2}\left(S_{K}^{\left(i\right)}\left(\theta_{0}\right)-\frac{K}{n}S_{n}^{\left(i\right)}\left(\theta_{0}\right)\right)+S_{K}^{*\left(i\right)}<0\}<\delta .$$

if *K* is large enough, so (3) is proven. The proof of (4) is the same by symmetry.

If we consider detecting multiple structural changes in the sequences, we can employ the binary segmentation method [30]. First use the single change test. If *H*_0_ is rejected, then find ${\hat{k}}^{*}\left(1\right)$ by (2). Next divide the sample into two subsamples $\left\{Y_{t},1\leq t\leq {\hat{k}}^{*}\left(1\right)\right\}$ and $\left\{Y_{t},{\hat{k}}^{*}\left(1\right)>t\leq n\right\}$, and test both subsamples for further changes. One continues this segmentation procedure until no subsamples contain further change-points.

## Simulation

To evaluate the finite sample performance of the proposed two test statistics (*W*_1_ and *W*_2_), we first simulate an ordinal time series {*Y*_*t*_} with *m* = 3 categories and length *n* = 100, 200, 500, 1000. The data are generated by

$$\begin{array}{ccc} \ln\frac{P\left(Y_{t}\leq 1\right)}{P\left(Y_{t}>1\right)} & = & \alpha_{1}-\beta_{1}\cos\left(2\pi t/12\right)-\beta_{2}Y_{\left(t-1\right)1}-\beta_{3}Y_{\left(t-1\right)2},\\ \ln\frac{P\left(Y_{t}\leq 2\right)}{P\left(Y_{t}>2\right)} & = & \alpha_{2}-\beta_{1}\cos\left(2\pi t/12\right)-\beta_{2}Y_{\left(t-1\right)1}-\beta_{3}Y_{\left(t-1\right)2}, \end{array}$$

where *α*_1_ = −0.5, *α*_2_ = 0.2, (*β*_1_, *β*_2_, *β*_3_)′ = (2, 0.5, 1)′, then the parameter vector ***θ*** = (*α*_1_, *α*_2_, *β*_1_, *β*_2_, *β*_3_)′. All simulation results are based on 1000 replications at the 0.05 significance level.

Suppose that we are only interested in *α*_1_ and *β*_1_, the others are nuisance parameters. Table 1 shows the empirical size of the two statistics under the null hypothesis *H*_0_. $W_{1}^{1}$ and $W_{1}^{2}$ denote the empirical size of *W*_1_ when testing for change in each of *α*_1_ and *β*_1_, respectively. *W*_1_ and *W*_2_ denote the empirical size of *W*_1_ and *W*_2_ when testing for change in both *α*_1_ and *β*_1_, respectively.

**Table 1:** The empirical size of *W*_1_ and *W*_2_ under the null hypothesis *H*_0_.

*n*	100	200	500	1000
$W_{1}^{1}$	0.013	0.014	0.018	0.019
$W_{1}^{2}$	0.01	0.018	0.02	0.014
*W*_1_	0.038	0.027	0.046	0.037
*W*_2_	0.038	0.045	0.033	0.047

It can be seen from Table 1 that the empirical size increases as the historical sample size *n* increases. When the sample size *n* = 1000, the empirical size of *W*_1_ and *W*_2_ is close to the significance level 0.05. In addition, based on the relation between the probability of type I errors when detecting *α*_1_ or *β*_1_ and the overall probability of type I errors, that is $W_{1}^{1}$, $W_{1}^{2}$ and *W*_1_ should satisfy $1-W_{1}=(1-W_{1}^{1})(1-W_{1}^{2})$. The results show that $1-W_{1}\approx (1-W_{1}^{1})(1-W_{1}^{2})$, which confirms the above inference.

**Fig 1:**
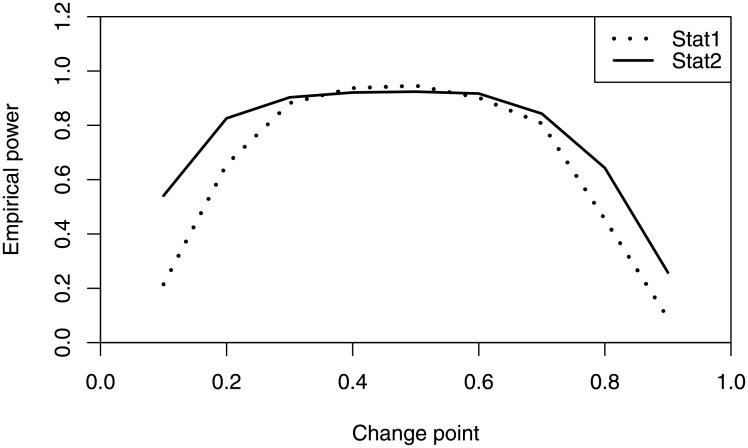
The empirical power of *W*_1_ and *W*_2_ under $H_{A}^{\left(1\right)}$ when k* = 100, 200, …, 900, *n* = 1000.

Under the alternative hypothesis *H*_*A*_, we consider the following three different situations:

$$\begin{array}{ccc} & & H_{A}^{\left(1\right)}:\alpha_{1}\;\text{changes}\;\text{from}\;-0.5\;\text{to}\;-1\;\text{at}\;k^{*},\;\beta_{1}\;\text{changes}\;\text{from}\;2\;\text{to}\;3\;\text{at}\;k^{*},\\ & & H_{A}^{\left(2\right)}:\alpha_{1}\;\text{changes}\;\text{from}\;-0.5\;\text{to}\;-1\;\text{at}\;k^{*},\\ & & H_{A}^{\left(3\right)}:\beta_{1}\;\text{changes}\;\text{from}\;2\;\text{to}\;3\;\text{at}\;k^{*}. \end{array}$$

where k* = 0.1*n*, …, 0.9*n*. Tables 2–4 summarize the empirical power of *W*_1_ and *W*_2_ under the alternative hypotheses $H_{A}^{\left(1\right)}$, $H_{A}^{\left(2\right)}$ and $H_{A}^{\left(3\right)}$ when k* = 0.1*n*, 0.5*n*, 0.8*n*. $W_{1}^{1}$ and $W_{1}^{2}$ denote the empirical power when testing for change in each of *α*_1_ and *β*_1_, respectively. *W*_1_ and *W*_2_ denote the empirical power of *W*_1_ and *W*_2_ when testing for change in both *α*_1_ and *β*_1_. From the simulation results, it can be seen that the empirical power of the two statistics increases with the sample size *n*, and is close to 1 when *n* = 1000. In addition, The empirical power of the two statistics varies according to different change-point locations, and reaches maximum when k* = 0.5*n*. Fig 1 describes the empirical power of the two statistics when k* = 0.1*n*, …, 0.9*n*. It is showed that the empirical power of *W*_1_ is higher than that of *W*_2_ when the change-point is located at the centre of the data, but *W*_2_ performs better when the change-point is located at the beginning or end of the data.

**Table 2:** The empirical power of *W*_1_ and *W*_2_ under the alternative hypothesis $H_{A}^{\left(1\right)}$.

	k* = 0.1*n*				k* = 0.5*n*				k* = 0.8*n*			
*n*	100	200	500	1000	100	200	500	1000	100	200	500	1000
$W_{1}^{1}$	0.016	0.016	0.032	0.057	0.055	0.092	0.292	0.6	0.027	0.04	0.094	0.208
$W_{1}^{2}$	0.01	0.028	0.089	0.14	0.069	0.186	0.502	0.859	0.027	0.042	0.112	0.33
*W*_1_	0.037	0.059	0.102	0.215	0.117	0.264	0.66	0.946	0.029	0.077	0.213	0.454
*W*_2_	0.084	0.153	0.3	0.541	0.068	0.168	0.569	0.924	0.03	0.064	0.259	0.643

**Table 3:** The empirical power of *W*_1_ and *W*_2_ under the alternative hypothesis $H_{A}^{\left(2\right)}$.

	k* = 0.1*n*				k* = 0.5*n*				k* = 0.8*n*			
*n*	100	200	500	1000	100	200	500	1000	100	200	500	1000
$W_{1}^{1}$	0.018	0.021	0.052	0.086	0.084	0.192	0.541	0.857	0.031	0.084	0.184	0.495
$W_{1}^{2}$	0.012	0.017	0.018	0.026	0.016	0.01	0.028	0.028	0.016	0.022	0.026	0.019
*W*_1_	0.028	0.044	0.082	0.098	0.104	0.201	0.545	0.873	0.053	0.067	0.215	0.498
*W*_2_	0.029	0.047	0.086	0.222	0.064	0.127	0.394	0.805	0.064	0.095	0.252	0.565

**Table 4:** The empirical power of *W*_1_ and *W*_2_ under the alternative hypothesis $H_{A}^{\left(3\right)}$.

	k* = 0.1*n*				k* = 0.5*n*				k* = 0.8*n*			
*n*	100	200	500	1000	100	200	500	1000	100	200	500	1000
$W_{1}^{1}$	0.006	0.017	0.019	0.029	0.013	0.026	0.029	0.037	0.011	0.015	0.02	0.029
$W_{1}^{2}$	0.02	0.031	0.077	0.158	0.059	0.19	0.599	0.901	0.014	0.039	0.13	0.415
*W*_1_	0.032	0.059	0.097	0.203	0.078	0.231	0.615	0.921	0.037	0.061	0.178	0.433
*W*_2_	0.1	0.142	0.265	0.436	0.052	0.142	0.461	0.813	0.022	0.036	0.145	0.429

In simulation for Table 2 both *α*_1_ and *β*_1_ change, whereas in Tables 3 and 4 only *α*_1_ and *β*_1_ changes at different change-points. Tables 3 and 4 indicate that most power stems from the parameter that is changed, which means *W*_1_ that could not only detect change in parameters, but also find the reason for rejecting the null hypothesis.

## Application to real data

**Fig 2:**
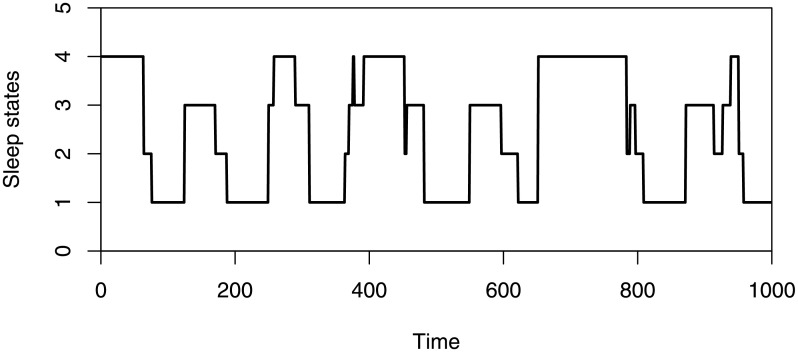
1000 sleep data (*Y*_*t*_) collected from the sleep state measurements of a newborn infant sampled every 30 seconds.

To illustrate the applicability of our results, we use 1000 sleep data (*Y*_*t*_) collected from the sleep state measurements of a newborn infant sampled every 30 seconds (Fokianos and Kedem [23]). The sleep states are classified as follows: (1) quiet sleep, (2) indeterminate sleep, (3) active sleep, (4) awake (Fig 2). According to the newborn’s sleep pattern, the sleep states have the following order: “(4)” < “(1)” < “(2)” < “(3)”, which means {*Y*_*t*_} is an ordinal time series. One goal of analyzing these data is to establish a correct model, and predict the sleep state based on the covariate information. Refer to example 6.3 of [23], ***Y***_*t*−1_ = (*Y*_(*t*−1)1_, *Y*_(*t*−1)2_, *Y*_(*t*−1)3_)′ is a significant predictor, which can be considered as a covariate. Then these data could be modeled by a cumulative logistic regression model

$$\begin{array}{c} \ln\frac{P\left(Y_{t}\leq 1\right)}{P\left(Y_{t}>1\right)}=\alpha_{1}+\beta_{1}Y_{\left(t-1\right)1}+\beta_{2}Y_{\left(t-1\right)2}+\beta_{3}Y_{\left(t-1\right)3},\\ \ln\frac{P\left(Y_{t}\leq 2\right)}{P\left(Y_{t}>2\right)}=\alpha_{2}+\beta_{1}Y_{\left(t-1\right)1}+\beta_{2}Y_{\left(t-1\right)2}+\beta_{3}Y_{\left(t-1\right)3},\\ \ln\frac{P\left(Y_{t}\leq 3\right)}{P\left(Y_{t}>3\right)}=\alpha_{3}+\beta_{1}Y_{\left(t-1\right)1}+\beta_{2}Y_{\left(t-1\right)2}+\beta_{3}Y_{\left(t-1\right)3}, \end{array}$$

where *α*_1_ = −14.722, *α*_2_ = −10.389, *α*_3_ = −4.078, *β*_1_ = 18.663, *β*_2_ = 12.173, *β*_3_ = 7.566.

**Fig 3:**
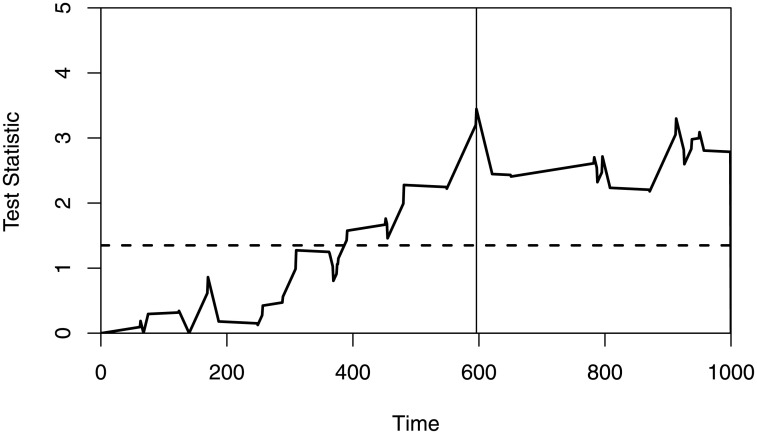
The value of *W*_1_ when testing for *α*_2_, the critical value at *α* = 0.05 is 1.35, and the location of change-point is 596.

Let ***θ*** = (*α*_1_, *α*_2_, *α*_3_, *β*_1_, *β*_2_, *β*_3_)′, then testing whether there exists a structural change in ***θ***, the result finds that a structural change occurs in ***θ*** by computing the test statistics *W*_1_ and *W*2. After this, using *W*_1_ to check which parameter occurs a structural change, the result shows that there exists a structural change in *α*_2_ at 596. Specifically, the maximum of *W*_1_ is 3.446, and the critical value is 1.35 when *p* = 1, *α* = 0.05, which gives a significant result (Fig 3). Re-estimate the parameters based on the first 596 samples and the last 404 samples, we have ${\hat{\alpha }}_{2}=-10.799$ for the former and ${\hat{\alpha }}_{2}=-8.57$ for the latter. We obtain AIC = 1646.65 for the adjusted model, and AIC = 1652.89 when assuming there is no change-point, which means to improve the model in some extent, so that we can make accurate predictions.

## Concluding remark

Cumulative logistic regression model is a generalized linear model, and has a wide application in health care. In this paper, two test statistics based on the efficient score vector are proposed to detect the structural change of cumulative logistic regression model. Under the null hypothesis of no change, we derive the asymptotic distribution of the two test statistics, and prove the consistency under the alternative hypothesis. Furthermore, we prove the consistency of the change-point estimator, and a binary segmentation procedure is provided for estimating the locations of possible multiple change-points. The finite sample performance is investigate by a monte carlo simulation, the results shows that the empirical size of the two statistics is close to the significance level 0.05, and the empirical power is approximate to 1 when the sample size is large. From the empirical power of view, the two test statistics have different advantages when the change-point occurs at different locations. Furthermore, the proposed statistic *W*_1_ could find the reason for rejecting the null hypothesis. Finally we apply the two test statistics to study 1000 sleep data collected from the sleep state measurements of a newborn infant sampled every 30 seconds, the results shows there exists a structural change in the model.

## Supporting information

S1 Table Simulation data for Table 1. (XLS)

S2 Table Simulation data for Table 2. (XLS)

S3 Table Simulation data for Table 3. (XLS)

S4 Table Simulation data for Table 4. (XLS)

S5 Table Simulation data for Fig 1. (XLS)

S6 Table 1000 sleep data for Fig 2. (XLS)

S7 Table Simulation data for Fig 3. (XLS)
